# Supplementary material for: Chiral Landau levels in Weyl semimetal NbAs with multiple topological carriers
Source: Nat Commun. 2018 May 10;9:1854. doi: 10.1038/s41467-018-04080-4 (PMC5945645; doi:10.1038/s41467-018-04080-4)
Supplement: Supplementary file 1 — Supplementary Information [file 41467_2018_4080_MOESM1_ESM.docx]

**Supplementary Note I. Sample composition**

**Supplementary Figure 1 | Atomic ratio determined by EDX.** A scanning electron microscope equipped with an energy-dispersive X-ray (EDX) spectrometer was employed to detect the element composition. Figure S1 shows nearly 1:1 atomic ratio of Nb and As. The slight deviation from the ideal ratio of 1 is close to equipment resolution. The stoichiometric composition and sharp X-ray diffraction peaks (Fig. 1b in the main text) ensure a good crystalline quality.

**Supplementary Note II. Magneto-infrared spectra for sample 2**

**Supplementary Figure 2 | Magneto-optical spectra for sample 2. a,** Normalized reflection rates at different magnetic fields. C3 is also observed. **b,** Extracted Landau level transition frequency. To compare with sample 1, all colored curves are copied from Fig. 2a in the main text, but the experimental data points in square are taken from sample 2. Error bars are added based on both experimental resolution and fitting uncertainty. **c**, Magneto-Drude-Lorentz fitting curve at 17.5T.

As shown in Supplementary Figure 2, the data from sample 2 show the same features as sample 1 in the main text. All the data analysis is quantitatively repeatable. From these two samples, there is no signature of Zeeman splitting in both magneto-optical spectroscopy and transport experiments. The intensity of M1 is higher than C2 at high fields, but has an opposite trend at low fields, suggesting different origins of the electronic bands. The low-field data points, however, were not used because the intensity of these peaks is fairly weak, yielding unreliable peak positions. Non-perfect fitting of C1, T1 and C2 suggests non-Lorentzian origin of the features. The features with the intensity order(s) of magnitude lower than C/T/M type peaks could be noise or contributed by other effects. Other than the intraband L_0_ to L_1_ transition, there also exists L_-1_ to L_0_ transition where the initial and final state are from the hole and particle band, respectively. These inter-band chiral Landau level transitions were not observed in the spectrum due to a much higher energy and the masking effect of normal Landau level transitions where a large density of states is contributed. However, the density of states changing from zero to positive value, combined with the finite impurity, results in the observation of C1 and C2. Generally, for low-Fermi-level material at high fields, the intensity of inter-band transition decreases with higher Landau level index. The highest index inter-band transition from which the frequency can be well extracted in both Weyl nodes is $L_{-4}\to L_{5}$/$L_{-5}\to L_{4}$. The labels for certain type of transitions are named as follows: “C” from *Chiral* Landau level, “T” from normal *Transition*, and “M” from *Multiple* carriers.

**Supplementary Note III. Zero-field reflection measurements**

**Supplementary Figure 3 | Reflection spectrum of NbAs.** a. Zero field reflectivity. b. Absolute reflection spectrum under different fields. Zero-field reflection spectrum was measured at liquid helium temperature on a combination of Bruke 113V and 80V (100-4000 cm^-1^). An *in-situ* overcoating technique was used as reference. As shown in Supplementary Figure 3, the reflectivity rapidly drops from 1 within 500 cm^-1^, exhibiting a typical Drude behavior. The reflection spectrum is multiplied by the normalized magneto-optical spectrum to yield the absolute reflection spectrum under a magnetic field. Since the detectable optical spectrum range is quite small in the high-field setup and the absolute reflectivity is far from zero (0.8 at 1000 cm^-1^), one cannot safely extract the optical conductivity through Kramers–Kronig relations. The absolute reflectivity is shown in Supplementary Figure 3b.

**Supplementary Note IV. Contributions of different electronic bands**

**Supplementary Figure 4 Summary of magneto-optical spectra from multiple carriers in NbAs.** There exist four bands including Weyl node 1 (red), Weyl node 2 (blue), inverted band (green), and classical band (grey). Solid lines are optical transitions involving the zeroth chiral Landau levels. The plots are identical to Fig. 2a in the main text but clearly present the origins of each optical transition.

**Supplementary Note V. Hall effect measurements**

**Supplementary Figure 5 | Hall effect measurements of NbAs. a,** Temperature-dependent Hall resistance. **b,** Hall resistance at 5K.

Hall effect measurements were carried out at different temperatures. *S*-shape feature can be witnessed from Supplementary Figure 5 showing the contributions from different kinds of carriers. The Hall effect is dominated by the hole carriers at high temperatures. In the low temperature regime (T<30 K), the Hall slope does not change with temperature. Quantum oscillations can be resolved at low temperatures. The measurements of the negative magneto-resistance have been well established in the NbAs system. The existence of the chiral magnetic effect cannot ensure the negative magneto-resistance near the quantum limit (requiring high magnetic field) due to the competition from the strong positive magneto-resistance. And the observation of negative magneto-resistance cannot directly be understood from chiral anomaly considering the extrinsic effects such as current jetting. In addition, if the magnetic field in the optical measurements can be extended to a higher value which makes L1 of W1 not intersect with the Fermi level, the C2 transition is anticipated to disappear as we observed in C3.

**Supplementary Note VI. Experimental challenge of probing chiral Landau level**

0

**Supplementary Figure 6 Intra-band transition edge frequency at constant magnetic field *B* in Weyl node based on Eq. (3) and (4).** A low Fermi level into the quantum limit kills the right edge. A little higher Fermi level quickly reduces the edge frequency and makes two edges merged together.

To observe the chiral Landau level at the experimental limit ($B<\text{17.5 T}$, $\omega>\text{100 cm}^{\text{-1}}$ in our case), the distance between Fermi level and the first Landau level is very important. A very low carrier concentration beyond the quantum limit results in the missing of the high energy edge as shown by the vertical dashed line. A higher carrier concentration could lead to a vanishing value of transition edge energy beyond the detection limit. For the Fermi level located between the first and second Landau level, there exist rich features. The inter-band transition edges for the Weyl node in the quantum limit do not exist. For those not in the quantum limit, they occur at higher energy where the normal inter-band transitions occur. Therefore, detecting both transition edges in Weyl semimetal requires strict conditions for magnetic field, sample’s Fermi energy and optical spectrum range. The Fermi level in this study is around 600 cm^-1^ and 200 cm^-1^ for two inequivalent Weyl nodes. For the Fermi level further close to the energy of Weyl point, the lower transition edge approximates the inter-Landau level transition in the 2D limit.

**Supplementary Note VII．Particle-hole asymmetry in NbAs**

Previous first-principle calculations reveal that NbAs is particle-hole asymmetric.[^1^](#_ENREF_1) The velocity for conduction and valence bands is different when moving away from the band crossing point. To capture the effect of particle-hole asymmetry, we added a diagonal term to the low-energy Hamiltonian that describes the Weyl fermions, as shown in the main text, by

$$H_{\alpha}(\mathbf{k})=\lambda_{\alpha}\sqrt{\eta_{1}^{2}k_{x}^{2}+\eta_{2}^{2}k_{y}^{2}+\eta_{3}^{2}k_{z}^{2}}+v_{\alpha}\mathbf{k}\cdot\sigma$$

where $\alpha\text{=}1\text{，}2$ corresponds to the Weyl node reaching or not reaching the quantum limit, and $\lambda_{\alpha}$ denotes the effect of asymmetry (either positive or negative) that changes the Fermi velocity oppositely for conduction and valence bands. We consider that a magnetic field is exerted in *z* direction, then the Landau level reads

$$E_{n,\alpha}(k_{z})=\left\{\begin{matrix} \lambda_{\alpha}f_{1}(n,B)+\mathrm{sgn}(n)v_{\alpha}\sqrt{(k_{z}-f_{2}(n,B))^{2}+2eB\left|n\right|},\left|n\right|\geq1 \\ \lambda\sqrt{eB}-v_{\alpha}k_{z},\text{ }n=0 \end{matrix}\right.$$

$f_{1}(n,B)=(\sqrt{(2n+1)eB}+\sqrt{(2n-1)eB})/2,f_{2}(n,B)=\lambda_{\alpha}(\sqrt{(2n+1)eB}-\sqrt{(2n-1)eB})/2v_{\alpha}$For the transition between the first Landau level and the chiral Landau level, we provide a more detailed analysis. For W1, we find that the Fermi level intersects with the particle-branch Landau levels, the frequency of the absorption edges can be calculated as follows. $E_{\text{F}}$ measures the energy offset between chemical potential and the Weyl node and $\omega_{0\to1,.1(2)}$ corresponds to the first (second) absorption edge. The energy of the first and second Landau level is given by

$$E_{1,\alpha}=\lambda_{\alpha}f_{1}(0,B)+v_{\alpha}\sqrt{[k_{z}-f_{2}(0,B)]^{2}+2eB}=\frac{(\sqrt{3}+1)\lambda_{\alpha}\sqrt{eB}}{2}+v _{\alpha}\sqrt{[k_{z}-\frac{(\sqrt{3}-1)\lambda_{\alpha}\sqrt{eB}}{2v_{\alpha}}]^{2}+2eB}$$

$$E_{0,\alpha}=\lambda_{\alpha}\sqrt{eB}-v_{\alpha}k_{z}$$

For $\omega_{0\to1,1}$, $\left|E_{F}\right|=E_{0,\alpha}=\lambda_{\alpha}\sqrt{eB}-v_{\alpha}k_{z}$ which gives

$$\begin{matrix} E_{1,\alpha}=\frac{(\sqrt{3}+1)}{2}\lambda_{\alpha}\sqrt{eB}+\sqrt{[\lambda_{\alpha}\sqrt{eB}-\left|E_{F}\right|-\frac{(\sqrt{3}-1)}{2}\lambda_{\alpha}\sqrt{eB}]^{2}+2v^{2}eB} \\ \text{ }=\frac{(\sqrt{3}+1)}{2}\lambda_{\alpha}\sqrt{eB}+\sqrt{[\left|E_{F}\right|-\frac{(3-\sqrt{3})}{2}\lambda_{\alpha}\sqrt{eB}]^{2}+2v^{2}eB} \end{matrix}$$

Thus,

$$\omega_{0\to1,1}=\frac{(\sqrt{3}+1)}{2}\lambda_{\alpha}\sqrt{eB}+\sqrt{[\left|E_{F}\right|-\frac{(3-\sqrt{3})}{2}\lambda_{\alpha}\sqrt{eB}]^{2}+2v^{2}eB}\text{-}\left|E_{F}\right|$$

C1 transition in the main text fits well to this equation. The extracted fitting parameters are also reasonable.

For $\omega_{0\to1,2}$,

$$\left|E_{\text{F}}\right|=E_{1,\alpha}=\frac{(\sqrt{3}+1)\lambda_{a}\sqrt{eB}}{2}+v_{\alpha}\sqrt{[\mathrm{k}_{z}-\frac{(\sqrt{3}-1)\lambda_{\alpha}\sqrt{eB}}{2v_{\alpha}}]^{2}+2eB}$$

which yields

$$v_{\alpha}k_{z}=\frac{(\sqrt{3}-1)\lambda_{a}\sqrt{eB}}{2}\pm\sqrt{[\left|E_{F}\right|-\frac{(\sqrt{3}+1)\lambda_{\alpha}\sqrt{eB}}{2}]^{2}-2v^{2}eB}$$

where the negative sign should be taken, thus

$$E_{0,\alpha}=\frac{(3-\sqrt{3})\lambda_{\alpha}\sqrt{eB}}{2}+\sqrt{[\left|E_{F}\right|-\frac{(\sqrt{3}+1)\lambda_{\alpha}\sqrt{eB}}{2}]^{2}+2v^{2}eB}$$

And

$$\omega_{0\to1,2}=\left|E_{F}\right|-E_{0,\alpha}=\left|E_{F}\right|-\frac{(3-\sqrt{3})\lambda_{\alpha}\sqrt{eB}}{2}-\sqrt{[\left|E_{F}\right|-\frac{(\sqrt{3}+1)\lambda_{\alpha}\sqrt{eB}}{2}]^{2}+2v^{2}eB}$$

C2 transition in the main text fits well to this equation with reasonable fitting parameters. T1 transition overlaps with the lower energy edge of W1.

From the schematic plots with particle-hole symmetry, C2 exhibits much lower energy than the experimental value. In fact, due to the much higher Fermi velocity from the asymmetry, the energy distance in particle band between Landau levels is enlarged. When approaching the quantum limit, the *k_z_* of optical transition edge moves towards zero leading to an accelerated increase of C2 transition energy at high fields.

The explicit expression of the energy for the first and second Landau level is derived as

$$E_{1}=\frac{(\sqrt{3}+1)\lambda_{\alpha}\sqrt{eB}}{2}+v_{\alpha}\sqrt{[\mathrm{k}_{z}-\frac{(\sqrt{3}-1)\lambda_{\alpha}\sqrt{eB}}{2v_{\alpha}}]^{2}+2eB}$$

$$E_{2}=\frac{(\sqrt{5}+\sqrt{3})\lambda_{\alpha}\sqrt{eB}}{2}+v_{\alpha}\sqrt{[\mathrm{k}_{z}-\frac{(\sqrt{5}-\sqrt{3})\lambda_{\alpha}\sqrt{eB}}{2v_{\alpha}}]^{2}+4eB}$$

As $\frac{(\sqrt{5}-\sqrt{3})\lambda_{\alpha}\sqrt{eB}}{2v_{\alpha}}-\frac{(\sqrt{3}-1)\lambda_{\alpha}\sqrt{eB}}{2v_{\alpha}}\approx0.1\lambda_{^{a}}\sqrt{eB}/v_{\alpha}$, when $\lambda_{\alpha}<v_{^{^{a}}}$, it is justified to neglect this difference, then

$$\omega_{1\to2}\approx\frac{(\sqrt{5}-1)\lambda_{\alpha}\sqrt{eB}}{2}+(2-\sqrt{2})v_{\alpha}\sqrt{eB}$$

which fits well to the T1 transitions.

Similarly,

$$\omega_{-1\to2}\approx\frac{(\sqrt{5}-1)\lambda_{\alpha}\sqrt{eB}}{2}+(2+\sqrt{2})v_{\alpha}\sqrt{eB}$$

$$\omega_{-2\to3}\approx\frac{(\sqrt{7}-\sqrt{3})\lambda_{\alpha}\sqrt{eB}}{2}+(2+\sqrt{6})v_{\alpha}\sqrt{eB}$$

$$\omega_{-3\to4}\approx\frac{(3-\sqrt{5})\lambda_{\alpha}\sqrt{eB}}{2}+(6+2\sqrt{2})v_{\alpha}\sqrt{eB}$$

Even for a large$\lambda\text{=}0.5v_{\alpha}$, the calculated right term is always one order of magnitude larger than the left term. For the higher index transition, the influence of the first term is even less and becomes negligible. For a large *n* and optical transitions with , the frequency can be approximated by

$\omega_{n_{i}\to n_{f},\alpha}=\lambda_{\alpha}(\sqrt{\left|n_{f}\right|}-\sqrt{\left|n_{i}\right|})\sqrt{2eB}+v_{\alpha}[\mathrm{sgn}(\mathrm{n}_{f})\sqrt{\left|n_{f}\right|}-\mathrm{sgn}(\mathrm{n}_{i})\sqrt{\left|n_{i}\right|}]\sqrt{2eB}$.

It is clear that the first term decreases rapidly with the increment of *n*, suggesting that the particle-hole asymmetry has a negligible impact on the inter-band transition with high index. The particle-hole asymmetry could contribute to the small intensity of low-index inter-band transitions (T2 and T3).

While we fit the infrared data to the model, we tried to keep the fitting parameters as few as possible. To describe all 10 C/T type features, only two parameters of Fermi velocity and asymmetry strength are required. Those fitted parameters agree well with previous photoemission experiments and DFT calculation.

**Supplementary Note VIII．Weyl semimetal without particle-hole asymmetry near the quantum limit**

The simplest two-band model of Weyl points gives

$$H(\mathbf{k})=v_{\text{F}}(k_{x}\sigma_{x}+k_{y}\sigma_{y}+k_{z}\sigma_{z})$$

With the introduction of magnetic field along *z* direction, the Landau levels are given by

$$E_{n,\pm}=\left\{\begin{matrix} \pm v_{\text{F}}\sqrt{k_{z}^{2}+2neB},n\geq1 \\ -v_{\text{F}}k_{z}\text{ }n=0 \end{matrix}\right.$$

For the system not in the quantum limit, there exist two intra-band transition edges. The lower frequency comes from the transition where the Fermi level crosses the chiral Landau level, therefore it reads

$$E_{0-1\text{，L}}=\sqrt{E_{\text{F}}^{2}+2v_{\text{F}}^{2}eB}\text{-}E_{\text{F}}$$

and the higher frequency comes from the transition where the Fermi level crosses the first Landau level, therefore it reads

$$E_{0-1\text{，H}}=E_{\text{F}}-\sqrt{E_{\text{F}}^{2}-2v_{\text{F}}^{2}eB}$$

Based on the schematic drawing of the optical transitions in Fig. 3e of the main text, one should also expect a transition between L_1_ and L_2_ at finite *k_z_* at crossing point of *E_F_* and L_1_. The transition energy $E_{1-2\text{,L}}$can be derived as follows:

$$E_{1}=v_{\text{F}}\sqrt{k_{z}^{2}+2eB}=E_{\text{F}}$$

$$E_{1-2,L}=E_{2}-E_{1}|_{E_{1}=E_{F}}=v_{\text{F}}\sqrt{k_{z}^{2}+4eB}-E_{\text{F}}=\sqrt{E_{F}^{2}+2ev_{\text{F}}^{2}B}-E_{\text{F}}$$

This is identical to $E_{0-1\text{，L}}=\sqrt{E_{\text{F}}^{2}+2v_{\text{F}}^{2}eB}\text{-}E_{\text{F}}$. Although this transition is also away from $k_{z}=0$, the optical transition does not include zeroth chiral Landau level, so we do not define it as C-type transition.

For the system in the quantum limit, however, the Fermi level does not cross the first Landau level, so the only observable feature comes from the low frequency edge which also follows Eq. (3). In table I of the main text, we use Eq. (3) and (4) for simplicity.

**Supplementary Note IX. Magneto-optical measurements in the mid-infrared range**

Similar to the far-infrared data in the main text, the mid-infrared spectra (Supplementary Figure 7) were also measured using the same experimental setup but with a different beam splitter. The data were also analyzed by the magneto-Drude-Lorentz fitting. The extracted Landau level transitions in the mid-infrared range all come from the massive trivial band (M-type in the main text) in NbAs with a finite band gap. The cyclotron mass of these topological trivial massive fermions in M3 and M4-M5 are 0.03*m_e_* and 0.07*m_e_*. where *m_e_* is free electron mass. The highest field of 17.5 T is limited by the superconducting magnet. A higher magnetic field can be achieved by using water-cooling magnet but the noise level needs to be reduced. We anticipate C2 to disappear under higher magnetic fields or with lower Fermi level samples as observed in C3.


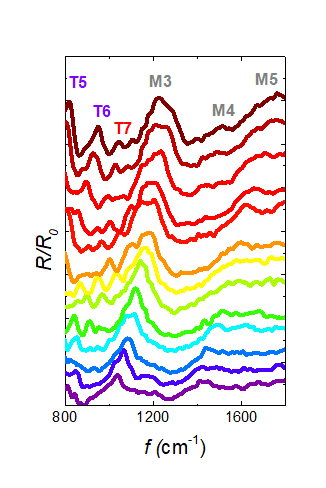


**Supplementary Figure 7 | Magneto-optical spectra in the mid-infrared range.** Both high frequency T transition and M transition can be observed.

**Supplementary Note X. Drude-Lorenz Fitting**

To quantitatively study the spectrum, we extracted the frequency of inter-Landau-level transitions and plotted it against the magnetic field. In a classical three-dimensional (3D) system, the Landau level transition induces a peak in the optical conductivity. Conductivity maxima do not necessarily locate at the same position of the reflection peaks. Therefore, in order to extrapolate the inter-Landau level transition energy, an absolute reflection spectrum is required. We performed the zero-field absolute reflection measurements using *in situ* gold coating technique at liquid helium temperature (Supplementary section III). The reflectivity quickly drops from 1 at 100 cm^-1^ to 0.8 at 700 cm^-1^ which is attributed to the free carrier Drude response. No phonon mode was observed in either the zero-field spectrum or the magneto-infrared spectra. Multiplying the normalized magneto-optical spectra with the zero-field data results in the absolute reflection spectra under magnetic fields. Then the experimental data were fitted to the Drude-Lorentz model in the presence of magnetic field[^2^](#_ENREF_2),

$$\sigma_{\pm}(\omega)=\frac{\omega}{4\pi i}(\varepsilon_{\infty}-1+\sum\frac{\omega_{p,n}^{2}}{\omega_{0,n}^{2}-\omega^{2}-i\gamma_{n}\omega\mp\omega_{c,n}\omega}),$$

where $\omega_{p,n},\omega_{0,n},\gamma_{n},\omega_{c,n}$ denote the plasma frequency, oscillator frequency, linewidth and cyclotron energy of the *n*-th oscillator, respectively. By performing the best fit, we can determine the cyclotron energy of the inter-Landau level resonances. The fitted curve shows a good consistency with the original experimental data. And the extracted cyclotron energy typically locates nearby but with a lower energy than where the apparent peak is positioned in the original data due to the difference between the conductivity maximum and reflectivity maximum. Also, the large background could influence the peak positions. The inter-Landau-level transition energy is obtained from the afore-mentioned fitting scheme. We cannot resolve a systematic change of peak linewidth probably due to the low peak intensity and small field range. Error bars are added based on both experimental resolution and fitting uncertainty. The error bars smaller than data symbols are not shown. The low-field data points beyond 8 T, however, were not used because the intensity of these peaks is fairly weak (suffering from the broadening effect), yielding unreliable fitted parameters. The model fits well to the experimental data at the whole field range as shown in Supplementary Figure 8.

**
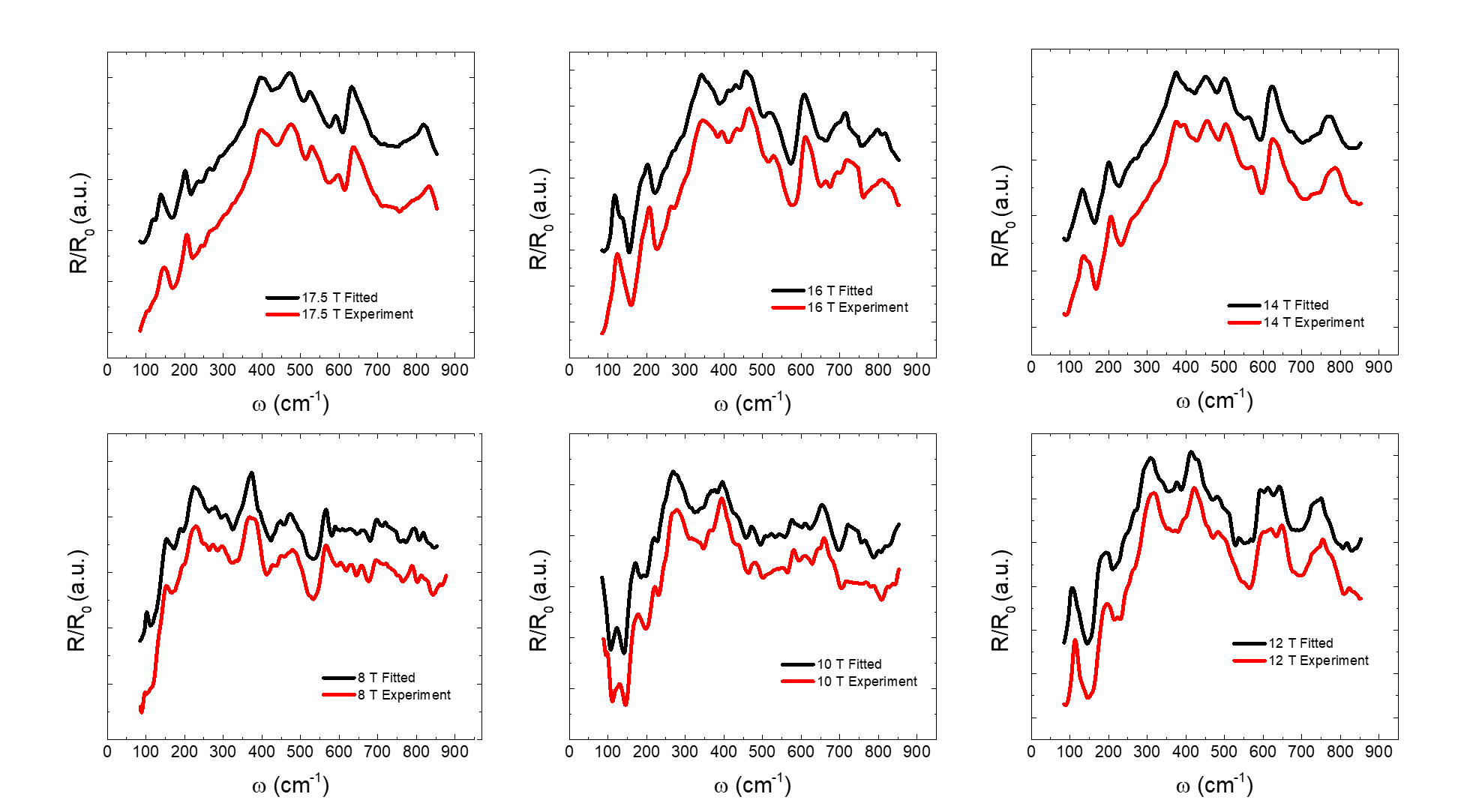
**

**Supplementary Figure 8| Drude-Lorentz fit at different magnetic field.**

**Supplementary Note XI. Landau quantization of massive fermions with or without band inversion in inversion-symmetry-breaking Weyl semimetals**

To understand M-type transitions, we have provided the main physics of an inversion-symmetry-breaking Weyl semimetal by a four-band minimal model whose form takes

$$H(k)=v_{x}k_{x}\tau_{x}\sigma_{z}+v_{y}k_{y}\tau_{y}+v_{z}k_{z}\tau_{x}\sigma_{x}+(m-Ck^{2})\tau_{z}+\Delta\tau_{y}\sigma_{x}$$

where $v_{x,y,z}$ represent the velocities, $m$ and $C$ are two constants, and $\Delta$ denotes the inversion-symmetry-breaking term. The energy spectra of this Hamiltonian are described by$E_{\pm,\pm}=\pm\sqrt{(\sqrt{v_{x}^{2}k_{x}^{2}+v_{y}^{2}k_{y}^{2}}\pm\left|\Delta\right|)^{2}+v_{z}^{2}k_{z}^{2}+(m-Ck^{2})^{2}}$. When $m\cdot C>0$, the band inversion takes place and Weyl points are located at the intersection of the two lines determined by $k_{x}^{2}+k_{y}^{2}=m/C$ and $\sqrt{v_{x}^{2}k_{x}^{2}+v_{y}^{2}k_{y}^{2}}=\left|\Delta\right|$ in the $k_{z}=0$ plane. Note that for this minimal model, $v_{x}\neq v_{y}$ is a necessary condition for the existence of Weyl points. However, here our focus is the magneto-optical physics induced by the gapped bands, thus we can take $v_{x}=v_{y}$ and only consider the two gapped bands $E_{\pm,+}$ for simplicity. With the assumption $v_{x}=v_{y}=v_{z}=v$ and the introduction of magnetic field in *z* direction, the Landau levels corresponding to the two gapped bands follow

$$E_{n}(k_{z})=\left\{\begin{matrix} \mathrm{sgn}(n)\sqrt{v^{2}k_{z}^{2}+(\sqrt{2v^{2}\left|n\right|eB}+\left|\Delta\right|)^{2}+m(n,k_{z})^{2}},\text{ }\left|n\right|\geq1 \\ \pm\sqrt{v^{2}k_{z}^{2}+\Delta^{2}+m(0,k_{z})^{2}},\text{ }\left|n\right|=0. \end{matrix}\right.$$

where $m(n,k_{z})=m-Ck_{z}^{2}-2eBC(\left|n\right|+\frac{1}{2})$. The observed band inversion has not been reported by other experimental approaches.

We argue that the slope ratio between M5 and M4 in fact agrees with the lowest two optical transition processes of the classical quadratic model. More explicitly, a simple normal insulator can be described by a quadratic Hamiltonian of the form $H=(\Delta_{0}+k^{2}/2m^{*})\sigma_{z}$, where the two eigenvalues of $\sigma_{\text{z},1}$ and -1, correspond to the conduction and valence bands, respectively. It is straightforward to find that the frequency of the [^1^](#_ENREF_1) lowest two inter-band transitions $L_{0^{-}}\to L_{1^{+}}$ and $L_{1^{-}}\to L_{2^{+}}$ with $\omega_{0,1}\text{=}2\Delta_{0}\text{+}2eB/m^{*}$ and $\omega_{1,2}\text{=}2\Delta_{0}\text{+4}eB/m^{*}$, respectively. It shows that the slope ratio is just 2. For M3, unfortunately we cannot make a definite statement whether it is originated from normal bands or inverted bands because there are no other spectra with the same zero-field intercept for further analysis. The above discussion is universal for other inversion-symmetry-breaking Weyl semimetals such as TaAs, where the similar features are expected.

**Supplementary Note XII. Landau quantization of two inequivalent Weyl fermions with different Fermi velocities**

T5 and T6 in the main text include transitions from both inequivalent Weyl nodes and therefore are labelled in purple. This overlapping feature is a natural consequence of different Fermi velocities from two inequivalent Weyl cones. As long as the ratio of Fermi velocity $v_{1}/v_{2}\leq1.3$ is satisfied in the two-Weyl-cone system (*n* is the transition index used in the main text), this overlapping of optical transitions will naturally occur because one can always find *n* to achieve$v_{1}/v_{2}\approx\frac{\sqrt{n+1}\text{+}\sqrt{n+2}}{\sqrt{n}+\sqrt{n+1}}$. In our case$v_{1}/v_{2}=1.15$, the inter-band Landau level transitions energy from L_-n_ to L_n+1_ in W1 and W2 thus reads

$$E_{1,n}=v_{1}(\sqrt{n}+\sqrt{n+1}\text{）}\sqrt{2ehB}$$

$$E_{2,n}=v_{2}(\sqrt{n}+\sqrt{n+1}\text{）}\sqrt{2ehB}$$

Note that

$\left.(\sqrt{n_{2}}+\sqrt{n_{2}+1})/(\sqrt{n_{1}}+\sqrt{n_{1}+1})\right|_{n_{1}=2,n_{2}=3}\text{=1.18}$,

$\left.(\sqrt{n_{2}}+\sqrt{n_{2}+1})/(\sqrt{n_{1}}+\sqrt{n_{1}+1})\right|_{n_{1}=3,n_{2}=4}\text{=1.13}$.

Therefore, $E_{1,2}\approx E_{2,3}$and $E_{1,3}\approx E_{2,4}$ describe the overlapping of T5 and T6, respectively. This is further supported by the intensity analysis. For all these peaks featured with overlapping transitions (T4/M2, T5 and T6), the intensity is generally higher than the adjacent ones without overlapping (T2, T3 and T7). The slope values of each transition and its transition index in Weyl node 1 (*n*_W1_) and Weyl node 2 (*n*_W2_) are given below.

| *n*_W2_ | *n*_W1_ | Slope ratio (cm^-1^B^-1/2^) |
| --- | --- | --- |
| 1 |  | 120.4 |
|  | 1 | 137.3 |
| 2 |  | 155.6 |
| 3 | 2 | 184.7 |
| 4 | 3 | 211.9 |
|  | 4 | 244.0 |

**Supplementary Note XIII. The overall Hamiltonian in NbAs**

A minimal Hamiltonian which simultaneously manifests the existence of Weyl points, inversion symmetry breaking, and particle-hole asymmetry takes the form

$$H(\mathrm{k})=\varepsilon_{0}(\mathrm{k})+v_{x}k_{x}\tau_{x}\sigma_{x}+v_{y}k_{y}\tau_{y}+v_{z}k_{z}\tau_{x}\sigma_{x}+(m-Ck^{2})\tau_{z}+\Delta\tau_{y}\sigma_{x}$$

where the first diagonal term breaks the particle-hole symmetry, and the last term breaks the inversion symmetry which is crucial for the existence of the Weyl points in a time-reversal invariant system. By making a unitary transformation, with

where , it is found that the Hamiltonian is decomposed at the direct sum of two parts, i.e., $\tilde{H}(\mathrm{k})=H_{1}(\mathrm{k})\oplus H_{2}(\mathrm{k})$ with

$$H_{1}(\mathrm{k})=\varepsilon_{0}(\mathrm{k})+\left(\begin{matrix} m-Ck^{2} & vk_{z}-i(\Delta-\sqrt{v_{x}^{2}k_{x}^{2}+v_{y}^{2}k_{y}^{2}}) \\ vk_{z}+i(\Delta-\sqrt{v_{x}^{2}k_{x}^{2}+v_{y}^{2}k_{y}^{2}}) & -m+Ck^{2} \end{matrix}\right)$$

the low-energy part which captures the physics of Weyl points, and

$$H_{2}(\mathrm{k})=\varepsilon_{0}(\mathrm{k})+\left(\begin{matrix} -m+Ck^{2} & vk_{z}+i(\Delta+\sqrt{v_{x}^{2}k_{x}^{2}+v_{y}^{2}k_{y}^{2}}) \\ vk_{z}-i(\Delta+\sqrt{v_{x}^{2}k_{x}^{2}+v_{y}^{2}k_{y}^{2}}) & m-Ck^{2} \end{matrix}\right)$$

the high-energy part which captures the physics of the gapped bands.

It is readily seen from the energy spectra of the $H_{1}(\mathrm{k})$ that the Weyl points exist when $v_{x}\neq v_{y}$ and the two closed rings, $C(k_{x}^{2}+k_{y}^{2})=m$ and $\sqrt{v_{x}^{2}k_{x}^{2}+v_{y}^{2}k_{y}^{2}}=\Delta$, are intersected. However, when $v_{x}\neq v_{y}$, the Landau level cannot be analytically obtained, which makes the analysis quite cumbersome. Thus, for a simple understanding of the main picture of the magneto-optical physics of Weyl points, a better choice is the low-energy two-band Hamiltonian expanded at the Weyl points (we have considered it in the main text), instead of $H_{1}(\mathrm{k})$ which also contains information far away from the Weyl points.

For the two gapped bands, as there exists an intrinsic energy scale (the Dirac mass), making the assumption $v_{x}=v_{y}$will have quite limited impact to the results. Thus, for the gapped bands, their corresponding Landau levels can be obtained by first solving the Landau levels of the full Hamiltonian $H(\mathrm{k})$ under the assumption $v_{x}=v_{y}$ and then choosing the two high energy branches. Also, due to the mass term providing an energy scale, the effect of the diagonal term to the optical transitions between the gapped bands are negligible and thus can be neglected.

Applying strong magnetic field could generate a gap in Dirac or Weyl bands due to the time-reversal symmetry breaking or crystal symmetry breaking. But based on our results, no signatures of gap opening were observed within 17.5 T probably because the field is not strong enough to break the symmetry and to induce a gap in Weyl bands. Therefore, we do not include field-induced gap in Hamiltonian.

In short, all the forms of the Hamiltonians in the main text can be derived from this general Hamiltonian under certain assumptions, and their forms are much more convenient for analysis in the regime where they are valid.

**Supplementary Note XIV. Lower edge of chiral Landau level transition and intra-band transition**

For both Weyl nodes, the frequency for 1-to-2 transition and 0-to-1 transition (the low-frequency edge) is very close when system is near the quantum limit (which can be seen from Figure 5 of the maintext and supplementary section VIII.). For system not reaching quantum limit (W1, or W2 with B<12T), the density of states for 1-to-2 transition is higher than 0-to-1 transition, the signature for the 0-to-1 transition will be overwhelmed by the peak arisen from the 1-to-2 transition. However, when quantum limit is reached (B>12T, for W2), the 1-to-2 intraband transition disappears, then the signature for the 0-to-1 transition will become sharp. As before the W2 entering the quantum limit, the frequency for 1-to-2 transition and 0-to-1 transition are very close, thus, the evolution of the frequency will not exhibit abrupt change, which is consistent with the experimental observation. From Fig. 1d, we can also see that for C1, the width of the peak in the low-field regime (e.g. B=8T) is much smaller than in the high-field regime (e.g. B=17T), this is consistent with our conclusion. As shown in the next section, the finite scattering could induce comparable amplitude between 1-to-2 and 0-to-1 transitions. Therefore, it is reasonable to find that the amplitude of C1 does not experience a sudden change when W2 reaches the quantum limit.

**Supplementary Note XV. Transition edge enhanced by scattering**

In the presence of impurity scattering, the edge for the transition involving chiral Landau level will be enhanced and the peaks for inter-band transitions will be suppressed (see blue dashed lines from Fig.6 in reference 3).[^3^](#_ENREF_3) Thus, it is reasonable to expect that the peak due to the transition edge has a similar feature with comparable amplitude as the feature from inter-band transitions.

**Reference**

1. Lee C-C*, et al.* Fermi surface interconnectivity and topology in Weyl fermion semimetals TaAs, TaP, NbAs, and NbP. *Physical Review B* **92**, (2015).

2. Lax B, Mavroides JG. Chapter 8 Interband Magnetooptical Effects. *Semiconductors & Semimetals* **3**, 321-401 (1967).

3. Ashby PEC, Carbotte JP. Magneto-optical conductivity of Weyl semimetals. *Physical Review B* **87**, 245131 (2013).
